# Genome-wide association with bone mass and geometry in the Framingham Heart Study

**DOI:** 10.1186/1471-2350-8-S1-S14

**Published:** 2007-09-19

**Authors:** Douglas P Kiel, Serkalem Demissie, Josée Dupuis, Kathryn L Lunetta, Joanne M Murabito, David Karasik

**Affiliations:** 1 Hebrew SeniorLife Institute for Aging Research and Harvard Medical School, Boston, MA, USA; 2 Department of Biostatistics, Boston University School of Public Health, Boston, MA, USA; 3 Section of General Internal Medicine, Boston University School of Medicine, Boston, MA, USA; 4 The National Heart Lung and Blood Institute's Framingham Heart Study, Framingham, MA, USA

## Abstract

**Background:**

Osteoporosis is characterized by low bone mass and compromised bone structure, heritable traits that contribute to fracture risk. There have been no genome-wide association and linkage studies for these traits using high-density genotyping platforms.

**Methods:**

We used the Affymetrix 100K SNP GeneChip marker set in the Framingham Heart Study (FHS) to examine genetic associations with ten primary quantitative traits: bone mineral density (BMD), calcaneal ultrasound, and geometric indices of the hip. To test associations with multivariable-adjusted residual trait values, we used additive generalized estimating equation (GEE) and family-based association tests (FBAT) models within each sex as well as sexes combined. We evaluated 70,987 autosomal SNPs with genotypic call rates ≥80%, HWE p ≥ 0.001, and MAF ≥10% in up to 1141 phenotyped individuals (495 men and 646 women, mean age 62.5 yrs). Variance component linkage analysis was performed using 11,200 markers.

**Results:**

Heritability estimates for all bone phenotypes were 30–66%. LOD scores ≥3.0 were found on chromosomes 15 (1.5 LOD confidence interval: 51,336,679–58,934,236 bp) and 22 (35,890,398–48,603,847 bp) for femoral shaft section modulus. The ten primary phenotypes had 12 associations with 100K SNPs in GEE models at p < 0.000001 and 2 associations in FBAT models at p < 0.000001. The 25 most significant p-values for GEE and FBAT were all less than 3.5 × 10^-6 ^and 2.5 × 10^-5^, respectively. Of the 40 top SNPs with the greatest numbers of significantly associated BMD traits (including femoral neck, trochanter, and lumbar spine), one half to two-thirds were in or near genes that have *not *previously been studied for osteoporosis. Notably, pleiotropic associations between BMD and bone geometric traits were uncommon. Evidence for association (FBAT or GEE p < 0.05) was observed for several SNPs in candidate genes for osteoporosis, such as rs1801133 in *MTHFR*; rs1884052 and rs3778099 in *ESR1*; rs4988300 in *LRP5*; rs2189480 in *VDR*; rs2075555 in *COLIA1*; rs10519297 and rs2008691 in *CYP19*, as well as SNPs in *PPARG *(rs10510418 and rs2938392) and *ANKH *(rs2454873 and rs379016). All GEE, FBAT and linkage results are provided as an open-access results resource at http://www.ncbi.nlm.nih.gov/projects/gap/cgi-bin/study.cgi?id=phs000007.

**Conclusion:**

The FHS 100K SNP project offers an unbiased genome-wide strategy to identify new candidate loci and to replicate previously suggested candidate genes for osteoporosis.

## Introduction

Osteoporosis is a skeletal disorder characterized by compromised bone strength leading to an increased risk of fracture [1]. In the United States alone, there are over 1.5 million fractures each year, including 280,000 hip fractures and 500,000 vertebral fractures. According to the recent U.S. Surgeon General's Report on Skeletal Health, fractures remain a large and growing public health concern [2]. Presently, the gold standard for assessment of fracture risk is measurement of bone mineral density (BMD, g/cm^2^) by dual-energy X-ray absorptiometry (DXA). Whereas low BMD is among the strongest risk factors for fracture [3,4], a number of clinical studies have demonstrated that other measurements, such as quantitative ultrasound (QUS) and bone geometry, are important for fracture prediction and osteoporosis treatment monitoring [5-7]. Thus, QUS of the calcaneus is associated with hip fracture, largely independent of BMD [8,9]. A growing body of evidence in recent years indicates that femoral geometry also contributes importantly to hip fracture risk [10,11].

BMD, QUS, and femoral bone geometry are approximately normally distributed, complex traits. A wealth of studies have documented BMD to be under strong genetic control with 50–70% heritability [12,13]. Similarly, QUS [8,14,15] and hip geometry [16-19] are probably regulated by additive genetic factors. However, despite years of research in the field of osteoporosis genetics, progress to date has been modest in successfully identifying major genes determining BMD, QUS, and bone geometry in the general population. The numbers of quantitative trait loci (QTLs) and genes linked and/or associated with osteoporosis-related phenotypes continue to expand and the list has become considerably more detailed and complex. More than 20 genome linkage scans to date have revealed multiple QTLs covering all chromosomes but the Y chromosome [20]. Moreover, the results from one study have inconsistently been replicated in other samples. To overcome these obstacles, a collaborative meta-analysis of 9 genome-wide linkage searches of BMD was recently conducted, including data from 11,842 subjects, members of 3,045 families [13]. The meta-analysis suggested a number of specific QTLs to be pursued further (1p13.3-q23.3 and 1q32-q42.3, 3p25.3-p22.1, 11p12-q13.3, 12q24.31-qter, and 18p11-q12.3). An additional factor that adds to the complexity of finding genes for osteoporosis-related traits is the notion (including our own [21,22]) that QTLs for bone density and geometry [23] are skeletal site-specific, age-group-, and sex-specific [24,25].

Numerous biological candidate genes for bone phenotypes have also been proposed, but few have been validated with large-scale evidence [13]. Recent meta-analyses for estrogen receptor alpha [26], collagen type I alpha 1 [27], methylenetetrahydrofolate reductase, [28] and vitamin D receptor [29] genes demonstrated that the polymorphisms in these genes each explain a small percentage of the variation in BMD or fracture. With the near completion of the International HapMap Project and the rapid improvements in high throughput genotyping technology, the ultimate understanding of the genetic basis of osteoporosis may come from a genome-wide association approach in which the whole human genome is surveyed for common genetic variation in osteoporosis-related heritable quantitative traits such as BMD [19]. To further advance the field of skeletal genetics, efforts are now turning toward studies that are able to cover the vast majority of the genome with a dense set of SNPs, in order to identify the genes linked and/or associated with osteoporosis-related traits. We thus performed genome-wide linkage and association analyses using a dense genome scan in members of extended pedigrees from the Framingham Heart Study (FHS).

## Materials and methods

### Study sample

The Framingham Osteoporosis Study is an ancillary study of the Framingham Heart Study (see description of the FHS sample in the Overview paper [30]). Beginning at biennial examination 20 of the Original Cohort, bone mineral density (BMD) was obtained using single and dual photon absorptiometry. The Original Cohort participants underwent bone densitometry by DXA with a Lunar DPX-L (Lunar Corp., Madison, WI, USA) during their examination 22 (1992–1993). In order to maximize the sample size, we used DXA scans from examination 24 (in 1996–1997) for 31 Original Cohort members who did not have DXAs at examination 22. The Offspring Cohort was scanned with the same machine at their examination cycle 6 or 7 (between 1996 and 2001). Right calcaneal bone ultrasonography (QUS) was performed with a portable QUS device, the Sahara^® ^bone sonometer (Hologic, Inc., Waltham, MA), in members of the Offspring Cohort at their examination cycle 6/7 and the Original Cohort during their exam 24 assessment. There were 1141 phenotyped individuals, 245 from the Original Cohort (86 men and 159 women, mean age 77.5 yrs) and 896 Offspring Cohort participants (409 men and 487 women, mean age 58.5 yrs), belonging to 241 families.

### Phenotype definition and residual creation

Original Cohort participants had bone measures performed at either exam 22 or 24. Offspring Cohort participants had these measures obtained during a period that included part of the official Framingham exams 6 and 7. The following traits were studied: Bone mineral density (including femoral neck, FNBMD; trochanter, TRBMD; L2–L4 lumbar spine, LSBMD); quantitative ultrasound of the calcaneus (broadband ultrasound attenuation, BUA; speed of sound, SOS), and geometric indices of the hip from DXA (femoral neck-shaft angle, NSA; femoral neck length, NeckLeng; section modulus, Z, and width, W, at the "narrow neck", Neck, and the shaft, S, regions of the hip). In total, 10 traits are reported here. Details of the measurements and phenotype definition [15,23,31] as well as coefficients of variation for the different component variables were previously reported for BUA (5.3%), SOS (0.4%) [32], LSBMD (0.9%), FNBMD (1.7%), TRBMD (2.5%) [33], and for the geometric traits, ranged from 3.3% (NeckW) to 9.1% (NeckLeng) [34].

Multivariate regression analysis was performed in each sex (men and women) and Cohort (Original and Offspring) in order to obtain normalized or ranked residual phenotypes, adjusted for covariates. Table 1 lists the covariates used for each trait. The covariate measurements have been described in previous work [22]. We used ranked residuals in order to correct for the deviations from the normal kurtosis and skewness in some of the phenotypes, since variance component analysis (VCA) is sensitive to the high kurtosis [35]. 

**Table 1:** Phenotypes included in the study

Trait	Abbreviation	Gender (N)	Cohort, exam cycle	Adjustment*
**Bone mineral density (BMD)**				
Femoral BMD**	FNBMD	Combined (1141)	Original, ex. 22–24, Offspring, ex. 6–7	1) multivariable-adjusted residuals (age, age^2^, height, BMI, smoking, physical activity, estrogen therapy)
	FNBMDf	Females (646)		
	FNBMDm	Males (495)		
Trochanter BMD**	TRBMD	Same as above	Same as above	Same as above
	TRBMDf			
	TRBMDm			
Lumbar spine BMD**	LSBMD	Combined (1117)	Same as above	Same as above
	LSBMDf	Females (641)		
	LSBMDm	Males (486)		
**Quantitative ultrasound (QUS)**				
Broadband ultrasound attenuation**	BUA	Combined (1105)	Original, ex. 24, Offspring, ex. 6–7	1) multivariable-adjusted residuals (age, age^2^, height, BMI, smoking, physical activity, estrogen therapy)
Speed of Sound	SOS	Combined (1104)	Same as above	Same as above
**Hip geometry**				
Neck-shaft angle**	NSA1	Combined (1096)	Original, ex. 22–24, Offspring, ex. 6–7	1) age, age^2^ 2) multivariable-adjusted ranked residuals (age, age^2^, height, BMI)
	NSAf	Females (622)		
	NSAm	Males (474)		
Femoral neck length**	NeckLeng1	Combined (1090)	Same as above	Same as above
	NeckLengf	Females (619)		
	NeckLengm	Males (471)		
Neck width**	NeckW1r	Combined (1095)	Same as above	Same as above
	NeckW1rf	Females (618)		
	NeckW1rm	Males (477)		
Neck section modulus**	NeckZ1r	Combined (1096)	Same as above	Same as above
	NeckZ1rf	Females (618)		
	NeckZ1rm	Males (478)		
Neck Cross-Sectional Moment of Inertia	NeckCSMI1r	Combined (1094)	Same as above	Same as above
	NeckCSMI1rf	Females (618)		
	NeckCSMI1rm	Males (476)		
Neck Average Buckling Ratio	NeckAvgBR1r	Combined (1096)	Same as above	Same as above
	NeckAvgBR1rf	Females (618)		
	NeckAvgBR1rm	Males (478)		
Shaft width**	ShaftW1	Combined (1028)	Same as above	Same as above
	ShaftW1f	Females (599)		
	ShaftW1m	Males (429)		
Shaft Section modulus**	ShaftZ1r	Combined (1028)	Same as above	Same as above
	ShaftZ1rf	Females (599)		
	ShaftZ1rm	Males (429)		
Shaft Cross-Sectional Moment of Inertia	ShaftCSMIr	Combined (1026)	Same as above	Same as above
	ShaftCSMIrf	Females (599)		
	ShaftCSMIrm	Males (427)		
Shaft Average Buckling Ratio	ShaftAvgBR1r	Combined (1028)	Same as above	Same as above
	ShaftAvgBR1rf	Females (599)		
	ShaftAvgBR1rm	Males (429)		
Inter-Trochanteric Average Buckling Ratio	ITAvgBR1r	Combined (1024)	Same as above	Same as above
	ITAvgBR1rf	Females (596)		
	ITAvgBR1rm	Males (428)		

*adjustment performed in each Cohort (generation) and gender

**primary traits reported here

### Genotyping methods

We used the Affymetrix 100K SNP GeneChip marker set in the Framingham Heart Study to examine genetic associations with the above phenotypes (see description of the FHS 100K and Marshfield STRs in the *Overview *paper [30]). We evaluated autosomal SNPs with genotypic call rates ≥80%, HWE p ≥ 0.001, MAF ≥10% in up to 1141 phenotyped individuals (495 men and 646 women). Ultimately, 70,987 SNPs were analyzed.

### Statistical analysis methods

We performed genome-wide association (GWA) analyses by two approaches, using both family-based and population-based methods. (See description of the general statistical methods for GWA and linkage analyses in the Overview paper [30]). Both sex-specific and combined-sexes analyses were performed. We used additive generalized estimating equation (GEE) and family-based association tests (FBAT) models to test associations of our phenotypes. Family based association testing has a great power to detect genetic variants of modest effect size [30,36].

In order to prioritize SNPs potentially associated with multiple phenotypes, we evaluated several phenotypic subgroups: (1) clinically important bone mineral density traits (FNBMD, TRBMD, LSBMD), (2) a combination of BMDs with BUA, (3) hip geometry (NSA, NeckLeng, NeckW and ShaftW), and (4) hip BMDs with hip geometry. We also focused on BMD in sex-specific subgroups. For each SNP we calculated the number of traits significantly associated with this SNP at alpha <0.01 in both GEE and FBAT, assuring that the FBAT and GEE effects were in the same direction. We then selected top SNPs with highest numbers of nominally significantly associated traits. For identical proportions of nominally significant traits, SNPs were additionally ranked by the mean of GEE p-values across traits (as well, GEE p-values were used for SNP ranking in the sex-specific subgroups due to a small sample size for FBAT analysis).

For SNPs that were selected by more than one strategy and that pertained to a specific gene (according to the NCBI Build 35), we perused *Entrez *Gene and also queried *PubMed *using the gene name and "osteoporosis", "fracture" or "bone mass" terms, in order to identify biological plausibility or evidence relating these genes specifically to bone disease.

Variance component analyses (VCA) for all phenotypes were performed on normalized or rank-normalized residuals using the computer package Sequential Oligogenic Linkage Analysis Routines (SOLAR, SFBR/NIH, San Antonio, TX) version 2.0, available online http://www.sfbr.org/solar/doc/00.contents.html. Heritability (h^2^) of each phenotype was estimated as the proportion of the total phenotypic variance attributable to the additive effects of genes. Subsequent linkage analyses were also conducted using SOLAR. This method, described in detail elsewhere [37] (see description of the linkage methods in the Overview paper [30]), entails specification of the genetic covariance between arbitrary relatives as a function of the identity-by-descent (IBD) relationships at a given marker locus and models the covariance matrix for a pedigree as the sum of the additive genetic covariance attributable to the QTL, the additive genetic covariance due to the effects of loci other than the QTL, and the variance due to unmeasured environmental factors. VCA linkage used IBDs values that were calculated from 11,200 markers.

## Results

Bone-related quantitative traits and derived phenotypes analyzed in the FHS 100K SNP resource are listed in Table 1. Each trait was analyzed as multivariable-adjusted residuals from cohort- and sex-specific models; in addition, geometric traits were adjusted for age, age^2 ^only. Among these, the following ten multivariable-adjusted residuals are the primary phenotypes presented here: BMD of the femoral neck (FNBMD; n with data = 1141), trochanter (TRBMD; n = 1141) and the second to fourth vertebral bodies of the lumbar spine (LSBMD; n = 1127); a quantitative ultrasound measure of the calcaneus, broadband ultrasound attenuation (BUA; n = 1105); femoral geometry measures at the "narrow neck" region of the hip and the femoral shaft including narrow neck section modulus (NeckZr; n = 1107) and width (NeckWr; n = 1106), femoral shaft section modulus (ShaftZr; n = 1037) and width (ShaftW; n = 1037), femoral neck-shaft angle (NSA; n = 1107), and femoral neck length (NeckLeng; n = 1101). Additional bone-related quantitative phenotypes are available in the FHS 100K SNP resource (web posted at http://www.ncbi.nlm.nih.gov/projects/gap/cgi-bin/study.cgi?id=phs000007) including calcaneal speed of sound; several hip geometry measures including neck cross-sectional moment of inertia; neck, shaft and intertrochanteric buckling ratio, and shaft cross sectional moment of inertia from these same examinations.

Heritability estimates for all bone phenotypes ranged from 30–66%. We consider here only multivariable-adjusted, residual phenotypes derived from ten primary traits defined above. There were 12 associations with 100K SNPs in GEE models at p < 0.000001 and 2 associations in FBAT models with p < 0.000001. The 25 SNPs with the lowest p-values in GEE (p < 3.5 × 10^-6^) and FBAT models (p < 2.5 × 10^-5^), ordered by chromosomal position, are shown in Tables 2a and 2b, respectively. Of these SNPs, 10 (GEE) and 19 (FBAT) were within 60 kb of a known gene. Several SNPs associated with phenotypes were in high pairwise LD (r^2 ^≥ 0.8). When this occurred we displayed results for the higher ranking SNP based on a combination of FBAT and GEE p-values. Of note, some SNPs were associated with more than one trait: thus, rs10514345 on chromosome 5 was associated (GEE) with NeckZ1 (combined sample) and LSBMD (females) and rs1209921 on chromosome 21 was associated (FBAT) with TRBMD (combined sample) and FNBMD (females) (Tables 2a and 2b, respectively).

**Table 2:** Top genetic associations with bone density and geometry based on the lowest p-value for GEE test (2a), FBAT (2b), and Linkage (2c) analyses

2a: Top 25 associations with bone density and geometry based on the lowest p-value of the GEE test						
**Phenotype **	**SNP**	**Chrom.**	**Physical Position**	**GEE p-value**	**FBAT p-value**	Gene region*

NeckZ1rf	rs6600671	1	120,812,532	**5.85 × 10^-7^**	3.18 × 10^-3^	
NeckZ1	rs2053506	3	19,350,795	**3.74 × 10^-6^**	2.68 × 10^-2^	KCNH8
TRBMDm	rs10510628	3	29,828,407	**2.83 × 10^-6^**	2.91 × 10^-2^	RBMS3
NeckW1rf	rs2054989	3	56,505,749	**6.06 × 10^-7^**	2.36 × 10^-3^	ERC2
ShaftW1f	rs922948	3	69,525,327	**1.75 × 10^-6^**	3.47 × 10^-3^	
ShaftW1	rs991258	3	103,256,733	**5.32 × 10^-7^**	3.12 × 10^-2^	
BUA	rs9291683	4	10,000,429	**2.07 × 10^-6^**	7.44 × 10^-4^	
NeckZ1rf	rs2548003	5	28,783,080	**2.40 × 10^-7^**	1.30 × 10^-1^	
NeckZ1rf	rs10515148	5	71,752,684	**6.10 × 10^-7^**	5.97 × 10^-3^	ZNF366
LSBMDf	rs10514345	5	90,460,035	**2.15 × 10^-6^**	9.85 × 10^-2^	GPR98
NeckZ1	Same as above			**1.81 × 10^-7^**	3.03 × 10^-2^	
NeckW1rf	rs4715166	6	13,324,037	**2.69 × 10^-6^**	9.97 × 10^-3^	PHACTR1
BUA	rs2214681	7	147,140,340	**2.72 × 10^-6^**	2.26 × 10^-2^	CNTNAP2
ShaftZ1R	rs10503887	8	31,752,989	**2.23 × 10^-7^**	1.77 × 10^-3^	NRG1
FNBMDm	rs2165468	10	3,506,105	**1.07 × 10^-6^**	6.71 × 10^-2^	
ShaftW1f	rs1452928	11	84,499,969	**7.10 × 10^-7^**	7.43 × 10^-3^	
NeckZ1rf	rs638882	11	115,269,255	**3.50 × 10^-6^**	6.33 × 10^-3^	
ShaftZ1rf	rs10492096	12	6,450,843	**3.35 × 10^-6^**	2.17 × 10^-3^	VAMP1
TRBMD	rs10506701	12	72,872,477	**1.40 × 10^-6^**	1.25 × 10^-1^	
ShaftW1f	rs10506821	12	78,999,391	**2.03 × 10^-7^**	1.80 × 10^-1^	
NeckZ1rm	rs1590305	13	36,933,189	**3.15 × 10^-6^**	2.21 × 10^-2^	
FNBMDm	rs9317284	13	62,532,351	**2.44 × 10^-7^**	2.86 × 10^-1^	
TRBMDf	rs4087296	16	80,935,282	**3.11 × 10^-7^**	6.18 × 10^-3^	
NSAm	rs4131805	18	24,313,110	**2.07 × 10^-6^**	1.87 × 10^-3^	
TRBMDf	rs4811196	20	35,903,108	**1.13 × 10^-6^**	7.03 × 10^-4^	CTNNBL1

**2b: Top 25 associations with bone density and geometry based on the lowest p value of the FBAT test**						

**Phenotype**	**SNP**	**Chrom.**	**Physical Position**	**GEE p-value**	**FBAT p-value**	**Gene region**

NeckW1rf	rs1395548	1	162,045,085	2.34 × 10^-3^	**1.22 × 10^-6^**	LMX1A
FNBMD	rs1538173	1	165,373,720	6.77 × 10^-2^	**2.80 × 10^-6^**	DPT
TRBMD	rs3762397	1	196,821,876	5.96 × 10^-3^	**2.78 × 10^-7^**	NR5A2
ShaftZ1rf	rs9287234	1	236,892,414	9.71\ × 10^-3^	**7.90 × 10^-6^**	FMN2
TRBMD	rs914951	1	239,278,119	1.82 × 10^-2^	**8.79 × 10^-6^**	
NeckW1	rs4143244	3	5,385,364	1.07 × 10^-2^	**7.05 × 10^-7^**	
NeckW1rf	rs1349205	3	54,093,157	2.22 × 10^-2^	**1.42 × 10^-5^**	CACNA2D3
ShaftW1	rs1870007	3	109,458,022	1.71 × 10^-1^	**1.32 × 10^-5^**	IFT57/HHLA2
FNBMDf	rs950649	3	121,567,065	7.63 × 10^-4^	**2.00 × 10^-5^**	FSTL1
NSA1	rs432446	4	127,906,277	2.84 × 10^-2^	**5.19 × 10^-6^**	
FNBMD	rs922028	4	181,162,903	9.55 × 10^-3^	**1.53 × 10^-5^**	
FNBMD	rs1823926	5	119,882,620	2.62 × 10^-1^	**1.27 × 10^-5^**	PRR16
NSA1	rs10503733	8	23,589,963	4.29 × 10^-4^	**1.79 × 10^-5^**	NKX3-1
NSAm	rs10503953	8	33,917,325	1.86 × 10^-3^	**1.32 × 10^-5^**	
NSAf	rs4876377	8	118,556,170	4.56 × 10^-2^	**1.93 × 10^-5^**	THRAP6
NSAf	rs10505600	8	133,882,045	2.48 × 10^-1^	**1.36 × 10^-5^**	PHF20L1
NSA1	rs1552896	9	14,831,387	4.02 × 10^-1^	**8.87 × 10^-6^**	FREM1
NeckZ1rm	rs7857590	9	18,979,893	1.36 × 10^-1^	**8.08 × 10^-6^**	C9orf138
BUA	rs700760	9	86,164,178	3.82 × 10^-2^	**1.42 × 10^-5^**	ZCCHC6
FNBMDf	rs2378731	9	87,175,111	5.53 × 10^-4^	**1.21 × 10^-5^**	
LSBMDm	rs10512315	9	103,334,877	1.70 × 10^-1^	**2.28 × 10^-5^**	
NeckW1rf	rs2048741	9	117,716,816	5.49 × 10^-3^	**2.40 × 10^-5^**	
ShaftZ1rm	rs6744	9	124,988,623	9.58 × 10^-2^	**1.05 × 10^-5^**	C9orf126
NeckLengf	rs773985	10	34,822,758	3.18 × 10^-2^	**1.05 × 10^-5^**	PARD3
NeckLengf	rs10490924	10	124,204,438	1.11 × 10^-1^	**1.80 × 10^-5^**	PLEKHA1/HTRA1
FNBMDm	rs10508076	13	101,270,253	5.41 × 10^-3^	**4.35 × 10^-6^**	FGF14
TRBMD	rs1865968	16	57,535,751	1.15 × 10^-1^	**1.72 × 10^-5^**	
TRBMD	rs1209921	21	39,078,145	2.51 × 10^-4^	**2.41 × 10^-5^**	C21orf24/ETS2
FNBMDf	Same as above			5.32 × 10^-5^	**6.65 × 10^-3^**	

**2c. Magnitude and Location of Peak LOD scores >2.5 for regions in the bone density and geometry phenotype group**						

**Phenotype**	**marker**	**Chrom.**	**physical location**	**maxLOD**	**1.5 LOD confidence interval**	

NeckLeng1	rs1759687	**1**	112,663,861	2.80	102,547,964–119,889,867	
ShaftW1	rs2320625	**2**	95,284,610	2.85	85,198,107–105,339,005	
NSA1	rs1304771	**2**	123,061,290	2.80	108,949,962–129,987,436	
BUA	rs842288	**3**	16,291,319	2.08	8,499,321–40,075,261	
NeckZ1	rs1386389	**4**	112,310,874	2.33	102,722,423–131,391,829	
NeckW1	rs2094541	**10**	130,314,425	2.56	128,465,158–135,165,518	
LSBMD	rs10492028	**12**	111,799,883	2.10	103,438,302–117,318,152	
ShaftZ1	rs10518923	**15**	55,573,538	3.02	51,336,679–58,934,236	
NeckW1	AFM049xd2	**16**	22,945,152	2.34	16,856,153–24,665,428	
BUA	rs10492922	**16**	27,519,162	2.20	26,434,120–50,596,704	
NeckZ1	rs6502901	**17**	5,864,832	2.37	451,209–8,829,083	
BUA	rs8081154	**17**	46,196,038	2.12	37,926,265–62,642,504	
NeckW1	rs750160	**20**	649,631	2.10	161,523–4,335,440	
ShaftZ1	rs7290139	**22**	44,039,280	3.01	35,890,398–48,603,847	

* SNP in a gene(s) or near (within 60 kb)

In Table 2c we present results with LOD scores above 2.0 from genome-wide linkage analyses with their corresponding 1.5-LOD confidence interval. There were linkage peaks observed for the majority of chromosomes, and in general there were a greater number of linkages with bone geometry traits than with either BMD or ultrasound measures. LOD scores ≥3.0 were found on chromosomes 15 (1.5 LOD support interval, 51,336,679–58,934,236 bp) and 22 (35,890,398–48,603,847 bp), with femoral shaft section modulus. There were no linkage regions of overlap with markers that were found to be associated with bone phenotypes in the FBAT and GEE analyses. Notably, when we restricted our sample to members of the Offspring Cohort, LOD scores for BMD phenotypes increased; for example, for LSBMD on chr. 7 (63,211,216 bp) from 1.25 to 2.43; for FNBMD on chr. 9 (104,660,212 bp) from 1.79 to 2.65, and on chr. 17 (63,038,023 bp) from 1.83 to 2.43 (not shown in the Table 2c).

In order to prioritize SNPs potentially associated with multiple phenotypes, we evaluated four phenotypic subgroups by both population- and family-based tests. The first subgroup was BMD (femoral neck, trochanter and spine), the second was BMD + bone ultrasound (BUA), the third was bone geometry (femoral neck-shaft angle, femoral neck length, femoral neck width, and shaft width), and the fourth, hip BMDs with hip geometry. For each SNP we calculated the number of traits in the subgroup that were significantly associated with the SNP at p < 0.01 in both GEE and FBAT, and then ranked SNPs with highest numbers of nominally significantly associated traits in which the direction of association was the same. In cases where SNPs had the same proportions of nominally significant traits, SNPs were additionally ranked by the mean of GEE p-values across traits.

In Table 3a we present only the SNPs selected based on associations with p-value of both GEE and FBAT < 0.01 with the first subgroup (BMD phenotypes only), for a combined sample of men and women. Using the annotation by UCSC genome browser tables http://genome.ucsc.edu/, of the top 20 SNPs for the BMD subgroup, 9 (45%) were identified in genes or within 60 kb of a known gene, and none were coding (Table 3a). The nominally significant intragenic SNPs were found in several genes, including cadherin 9 type 2 (*CDH9*) on 5p14 and gene deleted in colorectal carcinoma (*DCC*) on 18q21. It is also noteworthy that the association of TRBMD with rs1209926 on chr. 21 (39,078,994 bp) occurred in the region identified in our original genome-wide linkage study in the FHS [22] SNP rs2938392 in the peroxisome proliferator-activated receptor gamma (*PPARG*) was also associated with the BMD traits in GEE tests and less strongly in FBAT analyses (not shown in Table 3a).

**Table 3:** Combination of FBAT and GEE, for grouped BMD phenotypes*

3a: Combination of FBAT and GEE, for grouped BMD phenotypes in men and women combined							
**Chrom**	**Physical Position**	**SNP**	**Min p-value by GEE**	**Min p-value by FBAT**	% Significant P-values by GEE & FBAT**	**Function**	**Gene symbol**

1	177,489,285	rs7544774	3.34 × 10^-4^	7.55 × 10^-3^	67	intron	XPR1
1	231,181,475	rs7554650	1.67 × 10^-4^	5.93 × 10^-3^	67	unknown	
2	6,719,916	rs7584788	7.55 × 10^-3^	5.19 × 10^-4^	67	unknown	
2	15,963,228	rs2380707	1.14 × 10^-3^	5.22 × 10^-4^	67	unknown	
2	79,078,098	rs1261226	1.19 × 10^-5^	4.47 × 10^-3^	67	unknown	
4	177,742,191	rs9312601	2.22 × 10^-3^	7.03 × 10^-4^	67	unknown	
4	181,139,118	rs10520437	5.23 × 10^-4^	7.72 × 10^-5^	100	unknown	
4	181,162,903	rs922028	8.45 × 10^-4^	1.53 × 10^-5^	100	unknown	
5	26,917,842	rs1479679	1.13 × 10^-4^	2.38 × 10^-4^	67	intron	CDH9
5	53,527,854	rs16882423	1.65 × 10^-3^	2.38 × 10^-3^	67	intron	ARL15
7	9,739,152	rs1557978	1.73 × 10^-4^	3.97 × 10^-4^	33	intron	
7	11,301,740	rs10486135	1.67 × 10^-4^	5.95 × 10^-4^	67	intron	
7	18,183,021	rs10486301	1.94 × 10^-4^	9.43 × 10^-4^	67	unknown	
8	114,092,330	rs1156075	5.55 × 10^-3^	8.51 × 10^-3^	67	intron	CSMD3
11	95,219,586	rs546809	1.41 × 10^-3^	7.10 × 10^-5^	67	intron	MTMR2
12	103,526,872	rs10507180	7.18 × 10^-4^	4.16 × 10^-3^	100	intron	CHST11
16	76,268,225	rs8051539	2.08 × 10^-3^	5.80 × 10^-3^	67	unknown	
18	48,132,968	rs768207	1.94 × 10^-3^	2.99 × 10^-3^	67	intron	DCC
19	42,607,072	rs1465434	1.51 × 10^-3^	7.17 × 10^-4^	67	intron	ZNF569
21	39,078,994	rs1209926	2.51 × 10^-4^	3.21 × 10^-5^	33	unknown	C21orf24/ETS2

**3b: Sex-specific results for grouped BMD phenotypes^†^**							

**Chrom**	**Physical Position**	**SNP**	**Sex**	**Min p-value by GEE^‡^**	**Min p-value by FBAT^‡^**	**Function**	**Gene symbol**

1	48,261,693	rs560004	Males	8.91 × 10^-4^	3.48 × 10^-3^	unknown	
1	200,641,877	rs1935588	Females	6.14 × 10^-4^	7.62 × 10^-4^	unknown	
2	125,809,045	rs1215318	Males	1.23 × 10^-4^	2.96 × 10^-4^	unknown	
4	181,162,903	rs922028	Females	1.61 × 10^-4^	6.16 × 10^-3^	unknown	
5	26,917,842	rs1479679	Females	4.36 × 10^-5^	6.96 × 10^-3^	intron	CDH9
6	47,657,085	rs9296566	Females	2.27 × 10^-4^	5.08 × 10^-4^	intron	CD2AP
6	83,666,784	rs9294266	Males	5.32 × 10^-4^	2.01 × 10^-3^	intron	C6orf157
7	33,440,554	rs10486541	Males	1.54 × 10^-5^	1.95 × 10^-3^	unknown	
7	35,084,096	rs10486661	Females	9.34 × 10^-6^	9.98 × 10^-5^	unknown	TBX20
9	20,592,188	rs7867710	Females	6.96 × 10^-4^	2.97 × 10^-3^	intron	MLLT3
9	88,679,511	rs1779308	Males	3.09 × 10^-3^	4.94 × 10^-3^	unknown	
11	96,720,041	rs10501872	Females	4.03 × 10^-4^	1.02 × 10^-3^	unknown	
12	20,059,354	rs10505843	Females	2.02 × 10^-4^	7.38 × 10^-3^	unknown	
13	52,997,850	rs9316628	Females	2.51 × 10^-3^	5.22 × 10^-4^	unknown	
13	107,573,163	rs1223978	Males	3.42 × 10^-4^	5.07 × 10^-4^	unknown	
15	55,974,260	rs10518945	Females	6.19 × 10^-5^	6.15 × 10^-4^	unknown	ALDH1A2
17	11,054,857	rs2024134	Males	4.75 × 10^-3^	6.31 × 10^-5^	unknown	
17	53,546,007	rs181224	Males	2.15 × 10^-5^	2.90 × 10^-3^	unknown	Dlc2/OR4D1/OR4D2
18	48,132,968	rs768207	Females	1.35 × 10^-4^	2.57 × 10^-4^	intron	DCC
20	18,310,297	rs2328293	Females	7.74 × 10^-4^	4.35 × 10^-3^	locus	C20orf12

* Femoral neck, Trochanter, Lumbar spine BMD

** Percentage of traits nominally significantly associated with the SNP at p < 0.01 in both GEE and FBAT

^†^Subsamples with N < 400 excluded.

^‡^Percentage of traits nominally significantly associated at p < 0.01 in both GEE and FBAT was 67% for all SNPs.

We also examined men and women separately within the first phenotypic subgroup and confirmed the associations of BMD phenotypes, observed in the combined sample, with rs922028 (chromosome 4, no known gene), rs1479679 (in *CDH9*, chr. 5), and rs768207 (in *DCC*, chr. 18), all in women (Table 3b). There were also associations in one sex that were neither observed in the other sex nor in the combined sample, and men and women did not share many polymorphisms associated with BMD.

We also looked for evidence of pleiotropy by evaluating association results within the other three pre-specified phenotype subgroups, BMD + BUA and hip geometry, as well as combinations of hip BMD with hip geometry (fourth subgroup). Notably, pleiotropic associations between BMD and other traits were uncommon (not shown in a table). When considering BMD and BUA together (phenotype subgroup 2), there were nominally significant associations (p < 0.0005) with a SNP rs953934 in the interleukin 1 receptor-like 1 gene (*IL1RL1*) on 2q12 (not shown). Of the top SNPs for the Hip Geometry subgroup (NSA, NeckLeng, NeckW1r and ShaftW1), 50% were identified in genes or within 60 kb of a known gene; none were coding. The nominally significant intragenic SNPs were found in several genes, including rs7151976 in heat shock 70 kD protein 2 (*HSPA2*) on 14q24 and rs2834719 in runt-related transcription factor 1 (*RUNX1*, or *CBFA2*) on 21q22. Significant associations of SNPs with BMD phenotypes did not overlap with geometric phenotypes, and within the hip geometry subgroup, SNPs significant for one geometric phenotype did not overlap with other phenotypes within this subgroup (data not shown).

We further queried the FHS 100K array results, to verify, *in silico*, whether previously reported candidate genes for osteoporosis were associated with BMD, QUS and bone geometry in our sample. Thus Table 4 shows SNPs in these pre-defined candidate genes that were associated at p ≤ 0.05 in either GEE or FBAT models. We selected the following six previously reported genes that had been extensively studied: *COL1A1, CYP19, ESR1, LRP5, MTHFR*, and *VDR*. For the *MTHFR *gene, we found 1 non-synonymous coding SNP (rs1801133) that was associated with femoral shaft section modulus and neck-shaft angle by GEE and FBAT, respectively. We [38] have previously reported an association of this SNP with BMD in unrelated participants in the Framingham Osteoporosis Study and others have confirmed this association [28,39]. None of the three 100K SNPs in the *ESR1 *gene (rs1884052, rs3778099 and rs3866461) with nominally significant associations with bone phenotypes were in LD with widely published SNPs (rs2234693 and rs9340799). A single intronic 100K SNP from *LRP5 *(rs4988300) was associated with shaft section modulus in males and with femoral neck BMD in females. This 100K SNP was not in LD with published rs3736228 in *LRP5 *[40]. There were no LD data available on the *HapMap *website for the SNPs widely studied by others with respect to BMD (rs4988321 and rs627174). For the *VDR *gene there was a single 100K SNP (rs2189480) nominally significantly associated with femoral neck section modulus and spine BMD; however, there were no data available on LD between this 100K SNP and the 3 out of 5 SNPs reported in a recent meta-analysis of association studies for osteoporosis [29]. Two SNPs reported in that meta-analysis (rs1544410 and rs11568820) were not in LD with the one 100K SNP studied here. There were two 100K SNPs in the *CYP19 *gene (rs10519297 and rs2008691) associated with femoral neck-shaft angle that were in LD with at least one of the previously reported SNPs studied with respect to bone phenotypes (rs700518, rs4775936, rs10046, and rs11575899). Also, there was a single 100K SNP in the *COL1A1 *gene (rs2075555) that was associated with femoral neck width in women and shaft width in men; however, there were no data at the *HapMap *website regarding LD with the three other SNPs in this gene reported by other osteoporosis research groups (rs1107946, rs11327935, rs1800012).

**Table 4:** Associations of bone traits with selected candidate genes for osteoporosis with a FBAT or GEE p-value < 0.05

Chrom	Candidate Gene	Published SNP	Ref.	100K SNP	r^2^*	Physical Position	Lowest P-value by GEE	GEE Phenotype	Lowest P-value by FBAT	FBAT Phenotype
1	***MTHFR***	rs1801133	[38]	rs1801133	self	11,790,644	0.033	ShaftZ1rm	0.042	NSA1
			[39]							
			[28]							
6	***ESR1***	rs2234693	[45]	rs3866461	0.223	152,238,835	0.014	NSAm	**0.003**	NeckLengf
				rs1884052	0.004	152,383,480	**0.002**	ShaftZ1R	0.023	NeckW1rf
				rs3778099	0.002	152,510,689	**0.003**	BUA	0.019	FNBMD
		rs9340799	[45]							
11	***LRP5***	rs4988321	[40, 46]	rs4988300	no data	67,845,407	0.038	ShaftZ1rm	0.025	FNBMDf
		rs627174		rs4988300	no data					
		rs3736228	[40, 41]	rs4988300	0.030					
12	***VDR***	rs11568820	[29]	rs2189480	0.049	46,550,095	0.011	NeckZ1rm	0.021	LSBMD
		rs10735810	[29]	rs2189480	no data					
		rs1544410	[29]	rs2189480	0.045					
		rs7975232	[29]	rs2189480	no data					
		rs731236	[29]	rs2189480	no data					
15	***CYP19***	rs700518	[47]	rs10519297	0.902	49,328,952	**0.002**	NSA1	0.859	NSA1
			[48]	rs2008691	0.239	49,335,602	**0.003**	NSA1	0.012	NSA1
		rs4775936	[49]	rs10519297	0.58					
		rs10046		rs10519297	0.82					
		rs11575899		rs10519297	no data					
17	***COL1A1***	rs1107946	[50]	rs2075555	no data	45,629,290	0.034	NeckW1rf	0.010	ShaftWm
		rs11327935	[51]	rs2075555	no data					
		rs1800012	[52]	rs2075555	no data					

* r^2 ^between the 100K SNP and a corresponding published SNP (column to the left)

p-values < 0.005 are shown in bold.

## Discussion

*To our knowledge, this is the first genome-wide association study of many of the most commonly measured osteoporosis-related phenotypes. This unbiased approach allowed simultaneous exploration of multiple genetic loci rather than focusing on a single-gene association*. Using a strategy that combined GEE and FBAT association tests within the subgroup of BMD traits, 40 SNPs were each found to be associated with several bone phenotypes. More than half of these were in or near several genes, including cadherin 9 type 2 (*CDH9*) on 5p14, nuclear receptor subfamily 5, group A gene (*NR5A2*) at 1q32, gene deleted in colorectal carcinoma (*DCC*) on 18q21, and *PPARG *on 3p25. Some of the genes found to be associated with one or several bone density phenotypes have not previously been studied for osteoporosis. Analyses stratified by sex confirmed association of BMD phenotypes with *CDH9 *and *DCC *in women. The sex specificity of our findings is fully in line with the previous observations of both sex-specific linkage [13,21] and sex-specific genetic association with BMD [25,41].

Our phenotypic subgroup combining BMD and the ultrasound trait, BUA, demonstrated that the interleukin 1 receptor-like 1 (*IL1RL1*) gene on 2q12 was associated with all bone mass traits. In contrast there was a notable absence of pleiotropic associations between BMD and hip geometry (femoral neck length and width, neck-shaft angle, and shaft width). This is an important observation in light of the current debates about the role of femoral bone geometry in fracture prediction above and beyond BMD, as well as a criticism of DXA-derived bone geometric traits, which are perceived as partially captured in the BMD measurement. We have clearly shown that BMD traits (measured at the femoral neck, trochanter, and at the lumbar spine) share associations with SNPs in several genes, whereas there are no such SNPs that share associations with both hip BMD and hip geometric traits. Despite substantial shared genetic determinants between hip BMD and geometric traits (preliminary unpublished results from our group), these phenotypes are at least in part governed by distinct gene variants. Since genes contributing to variation in BMD do not always contribute to osteoporotic fractures [42], there is a necessity to genetically dissect geometric phenotypes along with BMD to derive a valid endophenotype of osteoporosis and to better encircle the complex heritability of this disease [31].

When we queried the FHS 100K association results, several SNPs in previously well-studied candidate genes for osteoporosis were identified (FBAT or GEE p < 0.05), such as rs1801133 in *MTHFR*; rs1884052 and rs3778099 in *ESR1*; rs4988300 in *LRP5*; rs2189480 in *VDR*; rs2075555 in *COLIA1*; rs10519297 and rs2008691 in *CYP19*. We also examined SNPs in other candidate genes of interest to our group such as SNPs in *PPARG *(rs10510418 and rs2938392) that were associated with BMD and ultrasound and SNPs in ANKH (rs2454873 and rs379016) that were associated with femoral neck section modulus.

We observed limited overlap between the results of the association and linkage analyses, which may, in part, be attributed to a low power of the linkage analysis. The resolution of linkage peaks depends not only upon the extracted information content of the genotyped markers, but also upon the useful recombination information contained in the sample (which is related to family structure and sample size). Despite low power for detecting modest genetic effects in linkage analyses, we identified several loci with a substantial indication of linkage, such as LOD scores ≥3.0 on chromosomes 15 (around 55,573,538 bp) and 22 (around 44,039,280 bp) for bone geometry phenotypes. Also, LOD scores for BMD phenotypes generally increased after restricting our sample to the members of the Offspring Cohort only, despite potential loss of power due to smaller sample size. This observation indirectly supports our previous finding of refined LOD scores in subgroups of age [21] and may be explained by the younger age of the Offspring Cohort members. The BMD phenotypes in that cohort might be less affected by aging-related artifacts in DXA scans, such as osteophytes, ossification of ligaments, and abdominal aortic calcification. We also observed little overlap between the results of the linkage analysis reported above and our previous studies of the same phenotypes [23,31], which may, in part, be attributed to low power of these linkage analyses and limited overlap between the 100K sample and the relatively large sample of FHS pedigrees published before [23,31].

To our knowledge, this is a first genome-wide association study of many of the most commonly measured osteoporosis-related phenotypes. It is distinct in many respects. First, this is a population-based sample rather than a study of affected families. Second, our study included adults from two generations of the same families. The longitudinal nature of the FHS and ongoing recruitment of the third generation (grandchildren of the Original Cohort) will enable us to validate the results of this study with an even larger sample of 3 generations. Third, results of this collaborative effort (FHS 100K SNP study) are publicly available on the web http://www.ncbi.nlm.nih.gov/projects/gap/cgi-bin/study.cgi?id=phs000007, which makes it a unique resource contributing to ongoing efforts of other groups.

One of the strengths of this study lies in the unbiased approach that allowed simultaneous exploration of multiple genetic loci rather than focusing on a single-gene association. However osteoporosis is likely to be a polygenic condition with a strong environmental component. Therefore, an obvious limitation of this study is the absence of analyses testing gene-environment and gene-by-gene interactions [43], which is a demanding element of study design and statistical analyses. We did not formally test for associations of the SNPs with some known environmental factors, such as smoking or estrogen replacement therapy in women, for which FHS has well-documented longitudinal data, because of sample size considerations.

A second potential limitation of this study is that there may be unmeasured factors that would affect the association or impact on generalizability of our results. Sampling of homogenous individuals is needed to minimize these effects. We attempted to approach this problem in part by stratifying our sample by gender; however, a more involved stratification, such as younger men or postmenopausal women, was not performed due to sample size restrictions. A third potential shortcoming of this study is the limited coverage of the genome by the 100K SNP array. These SNPs are undersampled from coding regions (both non-synonymous and synonymous) and oversampled from regions outside genes, relative to SNPs in the overall HapMap database [44]. Current plans of the FHS include the genotyping and analysis of over half a million genetic variants in approximately 9,000 men and women from three generations of the FHS. This higher density genotyping will provide wider coverage of genomic variation and thus will clearly have advantages for GWA and linkage studies in the future.

Fourth, at the present there is no clear-cut strategy for prioritization of the regions identified by our approach to whole genome analyses. We implemented one possible SNP prioritization strategy based on a somewhat arbitrary grouping of multiple phenotypes. Pleiotropy across different (but probably related) phenotypes may be an indication of genes that influence more than one biological pathway and thus may affect overall risk of osteoporosis.

Finally, we are hesitant to regard any result reported in this paper as genome-wide significant. Although observing evidence for association at a significance level of .0001 in both the GEE and FBAT test is very encouraging, it does not meet the standard of genome-wide significance. Not only do we have a large number of statistical tests for each phenotype, but we also have numerous phenotypes [30]. None of our findings should be considered true positive results until they are replicated in independent samples and/or functional studies are performed that provide the mechanism of the SNP effect.

## Conclusions and future directions

An unbiased genome-wide strategy to detect genetic associations with skeletal traits provides an opportunity to identify novel pathways for replication in other human populations. This study is thus hypothesis-generating and awaits replication in comparable samples. On one hand, ample data on genetic markers and associated traits may form the basis of new phenotype definitions, aimed at defining phenotypic groupings that are characterized by the sharing of genetic linkage and association. Such data may uncover biological mechanisms not obvious at the phenotypic level [42]. On the other hand, a composite genetic risk score that combines the effects of multiple loci may prove to be of more practical utility for a common and complex chronic disease such as osteoporosis, [43] rather than results of single-gene association studies. Identification of such a molecular profile and creation of an array for genetic screening will be a very useful tool for prevention and management of osteoporosis.

## Abbreviations

BMD = bone mineral density; Chr = chromosome; DXA = dual-energy X-ray absorptiometry; FBAT = family based association testing; GEE = generalized estimating equations; GWA = Genome wide association; HWE = Hardy-Weinberg equilibrium; IBD = identity-by-descent; LD = linkage disequilibrium; LOD = logarithm of the odds; MAF = Minor allele frequency; QUS = bone quantitative ultrasound; SNP = single nucleotide polymorphism; VCA = variance component analysis.

## Competing interests

The authors declare that they have no competing interests.

## Authors' contributions

All authors have made substantial contributions to conception and design or acquisition of phenotypic data. JD participated in the design of the study and performed statistical analyses; DPK, DEK, SD, JMM, and KLL contributed to the analysis and interpretation of data. DK and DPK drafted the manuscript and revised it critically for important intellectual content. All authors read and approved the final manuscript.
